# Mobile App (UPrEPU) to Monitor Adherence to Pre-exposure Prophylaxis in Men Who Have Sex With Men: Protocol for a User-Centered Approach to Mobile App Design and Development

**DOI:** 10.2196/20360

**Published:** 2020-12-01

**Authors:** Carol Strong, Huei-Jiuan Wu, Yuan-Chi Tseng, Chien-Wen Yuan, Yi-Fang Yu, Jay Chiehen Liao, Yi-Wen Chen, Yi-Chen Hung, Chia-Wen Li, Po-Hsien Huang, Nai-Ying Ko, Stephane Wen-Wei Ku

**Affiliations:** 1 Department of Public Health College of Medicine National Cheng Kung University Hospital Tainan City Taiwan; 2 Kirby Institute University of New South Wales Sydney Australia; 3 Institute of Service Science National Tsing Hua University Hsinchu City Taiwan; 4 Department of Industrial Engineering and Engineering Management National Tsing Hua University Hsinchu City Taiwan; 5 Graduate Institute of Library & Information Studies National Taiwan Normal University Taipei Taiwan; 6 Division of Infectious Diseases, Department of Internal Medicine College of Medicine National Cheng Kung University Hospital Tainan City Taiwan; 7 Department of Educational Psychology and Counseling National Taiwan Normal University Taipei Taiwan; 8 Department of Nursing College of Medicine National Cheng-Kung University and Hospital Tainan City Taiwan; 9 Taiwan Love and Hope Association Kaohsiung City Taiwan; 10 Division of Infectious Diseases Department of Medicine Taipei Veterans General Hospital Taipei Taiwan

**Keywords:** mobile apps, pre-exposure prophylaxis, event-driven, sexual behavior, men who have sex with men, user-centered design approach

## Abstract

**Background:**

Daily and on-demand pre-exposure prophylaxis (PrEP) has been well demonstrated to effectively prevent HIV acquisition for men who have sex with men (MSM). More than half of the MSM PrEP users in Taiwan prefer on-demand PrEP; however, on-demand PrEP involves a complicated dosing regimen because it requires precoital and postcoital dosing and sex events are hard to anticipate. Although there are a growing number of mobile apps designed to improve access to HIV prevention services and HIV medication adherence, few mobile apps focus on adherence to PrEP or are designed to accommodate a complicated, on-demand PrEP dosing schedule.

**Objective:**

The aim of this project is to evaluate the usability of a newly developed mobile app (UPrEPU) to assist MSM PrEP users to self-monitor their adherence to either daily or on-demand PrEP using a user-centered scheme.

**Methods:**

This research will be conducted in 2 phases: app development and usability study. In the app development phase, we will first conduct formative research with end users and stakeholders through in-depth interviews; the results will provide PrEP users’ and PrEP navigators’ personas as material used in the app conceptualization stage. PrEP navigators are individuals in the health care system that help HIV-negative individuals who need assistance in accessing PrEP care. A low-fidelity prototype of the app feature will be formatted by applying a participatory design approach to engage PrEP users, designers, and app developers in the design process of the app. Then, a high-fidelity prototype of the app will be developed for the usability study and refined iteratively by the multidisciplinary team and new internal testers. Internal testers include the research team consisting of experts in public health, infectious disease, and industrial design and a close network of the research team that is taking PrEP. In the usability study phase, we will enroll 70 MSM PrEP users and follow them up for 4 months. Usability, feasibility, and effectiveness of adherence monitoring will be evaluated.

**Results:**

Refinement of the UPrEPU app is currently ongoing. The usability study commenced in May 2020.

**Conclusions:**

The UPrEPU app is one of the first apps designed to help MSM PrEP users to self-manage their PrEP schedule better regardless of dosing modes. With a design-thinking approach and adapting to the cultural context in Taiwan’s MSM population, this novel app will have substantial potential to be acceptable and feasible and contribute to the reduction of new HIV infections.

**Trial Registration:**

ClinicalTrials.gov NCT04248790; https://clinicaltrials.gov/ct2/show/NCT04248790

**International Registered Report Identifier (IRRID):**

PRR1-10.2196/20360

## Introduction

Men who have sex with men (MSM) in Taiwan have been disproportionally affected by the HIV epidemic since 2006 [1]. Since 2015, approximately 2000 MSM per year have been newly diagnosed with HIV infections in Taiwan [1]. HIV biomedical prevention interventions, such as treatment as prevention and pre-exposure prophylaxis (PrEP), have been increasingly used in the past decade. PrEP has been well demonstrated in several clinical trials, and open-label studies have been conducted to provide high-level protection for both heterosexual and MSM against acquiring HIV [2-7]. PrEP has thus been included globally as one of the major components of the HIV prevention toolbox, including in World Health Organization guidelines [8,9], in the United States [10], and in the United Kingdom [11].

Daily PrEP — taking tenofovir disoproxil fumarate/emtricitabine (TDF/FTC) at the same time every day — has been recommended across genders and at-risk populations. For MSM, there is an alternative PrEP-dosing regimen: on-demand PrEP, referring to a double dose of TDF/FTC taken 2-24 hours before each sexual intercourse, followed by 2 single doses of TDF/FTC taken 24 and 48 hours after the first drug intake [12]. The IPERGAY study showed that on-demand PrEP in MSM was more efficacious than placebo administered to controls [7].

In France and the Netherlands, 23%-55% of PrEP users reported using on-demand PrEP [13,14]. Taiwan has a higher acceptance and willingness to use on-demand PrEP than daily PrEP [15]. Since PrEP was introduced in Taiwan in 2016, MSM have enjoyed the flexibility of choosing between daily and an on-demand PrEP schedule based on the recommendations from Taiwan’s National PrEP Guidelines [16]. A high proportion of Taiwanese MSM choose on-demand PrEP [17]. Based on real-world data, 56.7% of MSM report the use of on-demand PrEP at baseline and in 49.7% of follow-up visits [18]. PrEP users in Taiwan have various ways to access PrEP, including partially or fully subsidized by government-led demonstration projects, pay out of pocket for Truvada, or pay out of pocket for a generic brand of PrEP. Users are recommended to visit the clinics at least every 3 months for clinical measurements, laboratory measurements, and prescription refills based on Taiwan’s PrEP guidelines [16]. MSM in Taiwan have also been uncertain about communicating with sexual partners on social apps regarding their use of PrEP, thereby indicating their struggles with the social stigma related to PrEP [19].

The efficacy of PrEP relies heavily on adherence. The on-demand dosing regimen, however, can be complicated for most users because it requires that individuals anticipate sex in the next 2-24 hours and remember to take the pills before and after sex; therefore, adherence to on-demand PrEP can be hard to achieve.

Using mobile phone apps to seek sex partners has become common among MSM [20-23]. Adopting mobile phone apps to improve access to HIV prevention services and adherence to antiretroviral therapy among people living with HIV has grown increasingly popular [24-27]; however, only a few studies have focused on self-monitoring of PrEP adherence among MSM or were designed for daily dosing PrEP only [28,29], without meeting the growing need of on-demand PrEP and the possibility of switching between the 2 dosing regimens.

In response to the complexity of different dosing regimens and potential switching between the 2 dosing regimens, we developed a self-monitoring tool (UPrEPU app) to improve PrEP adherence regardless of the MSM user’s choice of dosing regimen and lifestyle. The aim of this study is to use a user-centered approach to evaluate the usability of this app. Users were involved at the beginning stage and throughout the development of this app to facilitate identifying users’ needs, enhancing participants’ engagement, and improving the app’s usability [30].

## Methods

### Study Design

This research protocol will be conducted in 2 phases: (1) app development phase, during which the app design and development process will be led by a multidisciplinary team with a user-centered design approach to ensure that the app will be acceptable and designed in line with PrEP users’ needs and context of use, and (2) usability study phase, during which we will evaluate the feasibility and usability of this app with a pilot sample.

#### Phase 1: App Development

The understanding stage will involve in-depth interviews with PrEP users and navigators as well as development of PrEP users’ personas and a journey map.

To identify PrEP users’ needs and the requirements of the mobile app–based PrEP adherence self-monitoring intervention, MSM who have experience using PrEP will be invited to participate in individual interviews. We will recruit a maximum of 30 participants from various routes, including the online LGBTQ community, PrEP clinics, and PrEP users’ online groups. Participants will undergo an in-depth online audio interview using a camera such as that available through Skype, Google Hangouts, or Line. The interview will explore the following: (1) PrEP users’ experience such as motivation, choices of PrEP-dosing regimens, and potential barriers to PrEP; (2) technologies used to self-manage adherence to PrEP, find sexual partners, and access PrEP-related information; (3) interactions between PrEP users and PrEP navigators.

PrEP navigators will also be invited to participate in individual interviews to understand their needs, usability requirements, and ideas related to this app. We will ask infectious disease physicians to refer PrEP navigators who are interested in participating in our study to contact us. The interviews will be online audio interviews conducted via Skype, Google Hangouts, or Line. During the interview, the following information will be obtained: (1) information related to the PrEP navigators’ routine work, (2) how they follow up with PrEP users and their dosing regimens, (3) tools they use to communicate with PrEP users at follow-ups, (4) challenges they encounter at follow-ups. All interviews will be audio-recorded for transcription and analysis.

Personas and users’ journey maps, a visualization of the process of an individual going through to reach a goal, are well-established user-centered approaches in user interface design to contribute to the creation of usable products, are able to provide an understanding of users for designers, and focus on users’ needs and pain points [31-33]. Several PrEP users’ personas and journey maps will be developed by the research team based on results from the previous stage. It can help developers and users visualize typical days in the PrEP users’ lives and PrEP managers’ daily routines. Personas will include demographics; goals, needs, and motivations; frustrations; and technologies they have applied to the PrEP-related issue. The journey maps will depict the PrEP service from the perspectives of PrEP users and PrEP navigators. In journey maps, the goals, actions, touch points, thoughts, emotions, and opportunities for stages — before, during, and after providing or receiving PrEP services — will be described. Personas and journey maps will be used in the upcoming participatory design workshop as an illustrative scenario for participants to generate shared understandings and design ideas [30].

During the app conceptualization stage, we will conduct a participatory design workshop (idea generation and prototyping) and develop a high-fidelity prototype.

The workshop aims to generate ideas for features of the UPrEPU app. We will apply a participatory design approach in the workshop to engage PrEP users, designers, and app developers in the app design process [34]. Designers include researchers with expertise in either human-computer interactions or public health. The participatory design approach between users and developers can help facilitate engagement and communication that focus on the users’ needs and search for technological solutions by brainstorming, exploring, and iteratively and cooperatively evaluating design ideas [35,36]. The workshop will be held in 1 day for 6 hours and will be held only once. The workshop will be led by a facilitator. In order to better generate ideas for app features, personas and journey maps developed from Phase 1 will be used in the workshop as an illustrative scenario to facilitate discussion and understanding for what PrEP users and navigators may encounter in daily life. A low-fidelity paper prototype of this app will be developed through the evolution and agreement of participants in the workshop.

A high-fidelity prototype of the UPrEPU app will be developed for both iOS and Android operation systems. During the development, the multidisciplinary team will work cooperatively to refine the prototype. After a full prototype is completed, new internal testers will access all features of the UPrEPU app to identify any remaining glitches, bugs, or usability concerns. Afterwards, a polished version of a final app prototype will be developed based on the feedback from the internal testers for an external usability study. We expect that the functions of the app may include sex and medication diary, medication adherence reminders based on users’ expected sexual behaviors and dosing regimens, education, and resources, although the final prototype will be developed based on the participatory design workshop.

#### Phase 2: Usability Study

After the internal test for the UPrEPU app is completed and optimized through feedback from the internal testers, we will evaluate the feasibility and usability of the app through a pilot study. This pilot study will also examine the preliminary effectiveness of the app in monitoring adherence to PrEP. We will enroll 70 MSM in 2 urban cities in Taiwan. Participants will be followed for 4 months and will complete an assessment at the beginning and every month. If they decide to join the study, participants will be asked to wear a wearable device that collects physiological sensor data.

Eligible participants will be HIV-negative men who meet the following criteria: (1) 20 years of age or older; (2) reside in Taiwan and able to understand, read, and speak Mandarin Chinese; (3) tested negative for HIV 3 months prior to enrollment for current PrEP users; (4) have laboratory results eligible to initiate PrEP based on Taiwan’s PrEP guidelines [16]; (5) currently taking PrEP or willing to initiate PrEP after enrollment; (6) report having ≥4 episodes of anal intercourse with men in the previous 1 month; (7) own an Android or Apple operating system (iOS) smartphone and are willing to download the study app; and (8) willing to wear the device the research team provides during the study period. Participants with the following characteristics will be excluded from the study: (1) have abnormal kidney function (creatinine clearance rate ≤60 mL/minute) or (2) currently on medications that might interact with PrEP, such as drugs containing lamivudine, at the baseline assessment. Participants who experience HIV seroconversion during the study period will be terminated from participation and referred to HIV care services. Their data will be still included in the analysis.

We will enroll participants from 2 medical centers in 2 major cities of Taiwan. Both medical centers have provided PrEP since 2016. Potential study participants will be referred by physicians and case navigators from the clinics. Their eligibility will be assessed by a screening tool available online. Afterwards, trained research assistants will inform those who are eligible about the purpose of the study and the information that will be collected in the study. Individuals who express interest in this study will be required to provide signed informed consent. For those who do not consent to participate, the reasons for declining participation will be documented. Eligible participants will download the UPrEPU app on their phone and answer a baseline questionnaire in the app regarding sociodemographics, mental health scales, sexual behaviors, and PrEP use at the baseline visit at the study site. Participants will also receive a wearable device that collects physiological sensor data. Participants will be encouraged to use all app components over the next 4 months and wear the wearable devices at all times.

Participants will be followed up monthly for the 4 months at the 2 medical centers where they were recruited. Each monthly visit will include rapid testing for HIV antigen and antibodies, measuring TDF/FTC concentration, and completing a follow-up questionnaire on their mental health scales, sexual behaviors, and PrEP use. Depression and anxiety will be assessed using the Patient Health Questionnaire-9 and General Anxiety Disorder-7, respectively. We collect these 2 mental health indicators since studies have shown that mental health status may be associated with adherence to medications [37].

Kidney function and sexually transmitted infections will be assessed at the end of the follow-up. The system usability scale for the UPrEPU app will be assessed during the first follow-up visit and at the end of the study. A face-to-face, semistructured qualitative interview will be conducted at each visit to assess the feasibility of the app. It will focus on any technical challenges participants may have encountered, recommendations for app improvement, and their satisfaction and comfort in using this app. All interviews will take around 30 minutes and will be audio-recorded for transcription and analysis.

Users can report bugs in the system and provide feedback in the app and to the customer service portal at any time. The bugs will be reported directly to our technology partners, whereas the issues reported and feedback will be documented, reviewed by the investigator team, and used in the next iterative stages of the app adaptation.

The primary outcomes include usability, feasibility, and effectiveness of adherence monitoring. Usability of the app will be measured using the system usability scale, a 10-item, 5-point Likert scale, which gives a global view of subjective assessment of usability [38]. A score above 50 out of 100 indicates acceptable [39].

The primary feasibility outcomes include frequency of app logins, use of app components such as PrEP-taking and sexual behavior reports, and the length of time the app is used based on the app analytics. Descriptive statistics will be used, and confidence intervals will be calculated to evaluate the primary feasibility outcomes. Secondary feasibility outcomes of this app will be analyzing data using thematic analysis methods from qualitative interviews focusing on technical challenges and satisfaction of the app during the follow-up [40].

We will examine the app users’ effectiveness of adherence monitoring with this app by comparing the pill-taking reports in the PrEP-taking diary component of the app with the tenofovir and emtricitabine drug concentrations in dried blood spot samples in the previous 7 days before the follow-up day. We will use correlation analysis to examine the consistency between the drug concentration in the dried blood spot samples and self-reported PrEP diary in the app. Higher correlation reflects higher effectiveness of adherence monitoring.

Participants will receive incentives for completing each follow-up visit and in-depth interview. This includes US $20 cash for each follow-up visit and US $33 cash for twice completing the in-depth interviews. Participants whose cumulative frequency of logins and use of features reach 90% will receive an additional US $33 at the end of the study.

### Trial Registration, Ethics, Consent, and Institutional Board Approval

The research and ethics presented in this study have been reviewed and approved by the Institutional Reviewer Board of National Cheng-Kung University in Tainan City, Taiwan (A-ER-107-337).

## Results

The usability study began enrollment in May 2020, and participants were followed up for 4 months. Study results will be available in 2021.

## Discussion

This paper provides an outline of a protocol for a research study that aims to evaluate the usability of a newly developed mobile app intervention using a user-centered scheme for self-monitoring adherence to either daily or on-demand PrEP.

The UPrEPU app is a novel smartphone app that accommodates the flexibility of switching dosing regimens and assists PrEP users adapt their dynamic lifestyles to better self-manage their PrEP taking, with the overarching goal of improving their adherence to PrEP. The UPrEPU app aims to be an acceptable, feasible, and capable tool for self-monitoring PrEP adherence in MSM populations.

Self-management of PrEP use and adherence to PrEP are particularly important during and after the COVID-19 pandemic. The COVID-19 pandemic may have had an impact on individuals’ ability to access HIV prevention medicine or treatment or may have limited access to health care services due to reduced hours [41]; in turn, MSM may be more likely to switch to an on-demand dosing regimen to cope with the possible shortage of pills.

A few mobile health interventions, with some of the most common features such as gamification, notifications, medication log, and education, target HIV prevention or PrEP adherence [28,42-45]. Our study is different from the rest of the mobile apps for PrEP adherence in its emphasis on incorporating sex behavior and medication logs to facilitate a more precise and personal reminder for whether the user has enough protection based on the correct dosage of PrEP. UPrEPU is the first app designed for both on-demand and daily dosing regimens for PrEP users, adopting a user-centered design. As the first app developed and operating in traditional Chinese focusing on PrEP adherence, we emphasized a participatory design throughout the development process to adapt to the cultural context in Taiwan’s MSM populations and that is likely in other Chinese-speaking communities around the world.

We anticipate a few limitations in the study protocol. First, we will only recruit individuals ≥20 years of age who are able to be consented legally. Therefore, our study results may not apply to younger MSM or adolescents since the usability or feasibility in such populations might be different. Also, the study might be more likely to recruit participants who have higher incomes and socioeconomic status based on the characteristics of the smartphone ownership eligibility criteria, which also affect the generalizability of the results. Second, the study requests that participants in phase 2 use the app and wear a portable device collecting physiological sensor data. Specifically, higher incentives will be received if participants use the app more frequently. We are likely to recruit highly motivated participants. The feasibility might be biased toward the higher end compared to when the app is released for download to the public in the future. Last, it is possible that providing a financial incentive for app use may impact participants’ behavior [46]; however, other factors such as social norms and intrinsic motivations may also influence participants’ behavior [47,48]. Our results from this pilot study will not completely reflect how people may use the app in real-world settings. Given that the effectiveness of PrEP depends on adherence and that switching between 2 PrEP dosing regimens is common and complex in real-world settings [18], the importance of obtaining related information and knowledge from this research is significant. The user-centered design approach can help us address the concerns regarding sensitive, private issues raised by marginalized populations. Findings from this study can be further used to design adaptive interventions to address different needs in various contexts and behavioral patterns. This study protocol describes the details of conceptualization of mobile apps and usability evaluation. Future studies could benefit from this study protocol by using a user-centered design approach in complex behaviors such as different drug-dosing regimens contingent on lifestyle behaviors to other mobile health interventions.

## Abbreviations

MSM: men who have sex with men
PrEP: pre-exposure prophylaxis
SUS: system usability scale
TDF/FTC: tenofovir disoproxil fumarate/emtricitabine
